# Vesical Tumours

**Published:** 1890-09-27

**Authors:** 


### VESICAL TUMOURS.

The prolonged experience of Sir Henry Thompson has induced him to publish a "classification of vesical tumours" (*British Medical Joural*). Sir H. Thompson commences with the most simple or the *mucous polypus*, which resembles polypus of the nasal cavity, although more solid and compact in structure. Hitherto, Sir Henry states, it has only been found in children. (2) *Simple myomatous tumours* are not very uncommon, but are unaccompanied by any characteristic signs. Their progress is slow, and the symptoms of papilloma, or of malignant growth are absent, but sometimes a papillomatous growth appears on the surface of the simple myomatous tumour. (3) *Papilloma* appears in two forms. The constituent element consists of a prominent fold of simple membrane supported by connective tissue, containing a large arterial twig as a central axis, while the outer surface of the membrane is closely covered by a layer of cylindrical epithelium. These growths were formerly termed "villous." When a considerable proportion of fibrous tissue is present, rendering the growth more solid, the designation "fibro-papilloma" is more appropriate. These growths form very slowly, but eventually give rise to repeated attacks of hæmorrhage. This symptom may be mistaken as indicating renal disease. Microscopic examination of the urine will probably detect specimens of the growth. Washing out the bladder with water, and scrutinizing the débris is a good plan. In two or three cases, when Sir Henry Thompson failed to discover specimens of the growth under the microscope, although symptoms pointed to papilloma, he introduced a light flat-headed lithotrite, and by opening and closing it slowly a few times removed a tiny fragment which, under the microscope, afforded the desired evidence. There are, however, other features of distinction from renal disease. The patient commences an act of micturition with clear urine, and at the end of the act bright florid blood is mixed with the stream, or appears alone at the close. When blood comes from the kidneys it is mixed with the urine, but bleeding may also take place from a malignant growth. "If for a considerable period of time there is still but little pain experienced, and the act of micturition is not very frequent, the bleeding is certainly not due to a sarcomatous or carcinomatous growth." Examination *per rectum* affords valuable information. Scirrhous growths are hard, sarcomata full and rounded. Papilloma is rarely recognisable by the rectum, or even by the sound, often manifesting its presence first by hæmorrhage. The other vesical growths mentioned by the author are (4) *Fibrous tumours*, which involve the deep structures of the bladder; (5) *Epithelioma*, the nature and characters of which in the bladder are those of the growth in all other parts of the body; (6) *Scirrhus or cancer*, not occurring till after middle life; (7) *The round and spindle-celled sarcomata*, formerly known as encephaloid.

Sir Henry Thompson gives a summary of the results of operation in 41 cases. In seven cases all papilloma, excepting one myoma, there has been no reappearance of symptoms of any kind, three years having elapsed since the most recent, and ten years since the first. In three cases the growth was removed by the forceps through a perinæal incision; in one female, dilatation of the urethra sufficed; and in three the suprapubic operation was performed. In fifteen other cases death occurred at different periods varying between three days and four months after the operation. Ten were the subjects of malignant growths. Two were papillomata, and the rest myomata. Nineteen remaining patients lived after the operation from a year up to four years.

Experience shows that the subject of a bleeding vesical tumour invariably dies if the malady is not interfered with, but experience also shows, that not unfrequently a cure is effected by the removal of a papillomatous tumour. But in all cases when the presence of a malignant tumour—carcinoma, sarcoma, epithelioma—can be ascertained, Sir Henry Thompson says it is useless to attempt to remove the growth. Still when there is doubt as to the nature of the malady, "it must almost invariably be our duty to recommend an operation for removal," but Sir Henry Thompson confesses that in some cases "not until the scalpel has opened the way and permitted the operator to lay his finger on the growth, and thus ascertain its physical character, can he say whether it can be completely removed." Moreover, it must not be forgotten that a papillomatous growth may be present as a secondary growth upon another form of tumour.

We have recently heard a great deal about the Leiter endoscope. Sir Henry Thompson states he has tested its value in its most improved form, and finds that the employment of the endoscope involves much irritation in some cases. That in the presence of a tumour which the slighest mechanical contact causes to bleed, the observation is an imperfect one. Sir Henry prefers "digital exploration" through a small perinæal section to the use of the endoscope. We fancy, however, most patients would prefer a trial of the endoscope as a diagnostic measure, before submitting to perinæal section.

